# Current Knowledge and Perspectives of Immunotherapies for Neuroblastoma

**DOI:** 10.3390/cancers16162865

**Published:** 2024-08-17

**Authors:** Chenkai Mao, Maria Poimenidou, Brian T. Craig

**Affiliations:** 1 Department of Surgery, Medical College of Wisconsin, Milwaukee, WI 53226, USA; cmao@mcw.edu; 2 Center for Immunology, Medical College of Wisconsin, Milwaukee, WI 53226, USA; mpoimenidou@mcw.edu

**Keywords:** neuroblastoma, immunosuppression, tumor microenvironment, immunotherapy

## Abstract

**Simple Summary:**

Neuroblastoma (NBL) is the most common extracranial cancer in children. High-risk NBL is characterized by bone marrow metastasis and *MYCN* oncogene amplification and is associated with a dismal five-year overall survival of approximately 50%, despite an intensive multimodal treatment regimen including chemotherapy, surgery, high-dose chemotherapy with autologous stem cell rescue, and radiation. Herein, we systemically reviewed contemporary therapy schemes and their challenges as well as recent advancement in effective treatments for NBL. Due to its relative rarity, we hope this review can serve as a useful guidebook and potentially provide new insights to researchers and clinicians interested in this field.

**Abstract:**

Neuroblastoma (NBL) cells highly express disialoganglioside GD2, which is restricted and weakly expressed in selected healthy cells, making it a desirable target of immunotherapy. Over the past two decades, application of dinutuximab, an anti-GD2 monoclonal antibody (mAb), has been one of the few new therapies to substantially improve outcomes to current levels. Given the persistent challenge of relapse and therapeutic resistance, there is an urgent need for new effective and tolerable treatment options for high-risk NBL. Recent breakthroughs in immune checkpoint inhibitor (ICI) therapeutics have not translated into high-risk NBL, like many other major pediatric solid tumors. Given the suppressed tumor microenvironment (TME), single ICIs like anti-CTLA4 and anti-PD1 have not demonstrated significant antitumor response rates. Meanwhile, emerging studies are reporting novel advancements in GD2-based therapies, targeted therapies, nanomedicines, and other immunotherapies such as adoptive transfer of natural killer (NK) cells and chimeric antigen receptors (CARs), and these hold interesting promise for the future of high-risk NBL patient care. Herein, we summarize the current state of the art in NBL therapeutic options and highlight the unique challenges posed by NBL that have limited the successful adoption of immune-modifying therapies. Through this review, we aim to direct the field’s attention to opportunities that may benefit from a combination immunotherapy strategy.

## 1. Introduction

Neuroblastoma (NBL) is one of the most common childhood cancers and is responsible for about 15% of all childhood cancer deaths [1]. Due to its unique sympathetic nervous origin, NBLs do not present with high tumor mutational burden in comparison to many adult solid tumors. This low mutation burden poses specific challenges for effective immunotherapies that have been successfully implemented in adult tumor treatment regimens [2,3]. Hence, treatments for NBL need to be tailored to the specific conditions present in the NBL tumor microenvironment (TME).

A classic feature of NBL is its high degree of heterogeneity, which complicates the diagnosis and choice of optimal treatment regimen. Primary NBL tumors can present at different locations, including neck, chest, abdomen, or pelvis, resulting in varying symptoms. Primary tumors located in the abdomen have higher probability of recurrence compared to other sites; however, primary tumor location is not a feature in the consensus risk stratification system utilized clinically, since other features such as age, stage, and biological features of the tumor provide much stronger prognostic value [4]. Additionally, nearly half of NBL patients present with metastases which most often involve the bone marrow, as well as lymph nodes, liver, and, occasionally, the lungs [5]. Newly diagnosed NBL patients are stratified based on the International Neuroblastoma Risk Group staging system including age, tumor stage, histology, and genetics into different risk groups including very low, low, intermediate, and high risk based on predicted risk of developing relapsed disease.

Among different risk groups of NBL, high-risk NBL is the most prevalent and most aggressive; the overall survival rate for these patients is less than 50% even with intensive multimodal treatment [4,6]. Nearly half of high-risk NBL patients carry amplification of the *MYCN* oncogene, a broad transcriptional activator that regulates multiple aspects of the tumor immune interaction. *MYCN*-amplified NBLs exhibit lower MHC-I with lower CD8+ T cell and higher regulatory T cell (Treg) infiltration [7,8]. Thus, it is critical to understand the contribution of *MYCN* status to disease pathogenesis and progression. Current understanding remains incomplete, as *MYCN* largely serves as a prognostic biomarker, and treatment is not currently adjusted for *MYCN* status independently from risk-group assignment.

**Figure 1:**
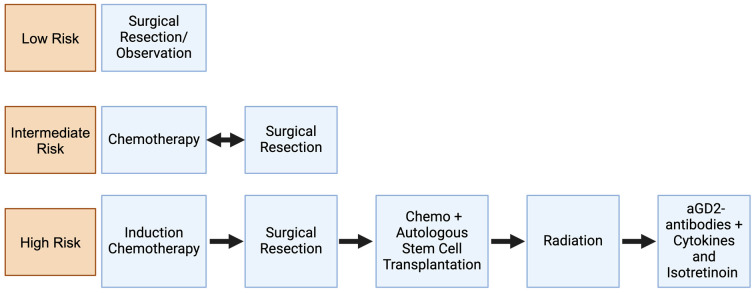
Overview of modern risk-adapted treatment regimen for neuroblastoma. These treatment schemes are tailored to an individual patient’s risk of relapse and include observation or surgery alone for the low-risk group; multiple rounds of chemotherapy with surgery for the intermediate risk group; and a multimodal strategy combining chemotherapy, surgery, high-dose chemotherapy with autologous stem cell rescue, radiation, and immunotherapy for the high-risk group. This figure was created with BioRender.com.

Modern treatment for NBL is highly dependent on the patient’s assigned risk group, with most recent efforts focused on de-escalating therapy for low-risk disease and optimizing the application of intensive multimodal therapy for high-risk patients [9,10,11,12,13]. These risk-adapted treatment schemes include observation or surgery alone for the low-risk group [12,14]; multiple rounds of chemotherapy with surgery in the intermediate risk group; and multimodal strategies combining chemotherapy, surgery, and high-dose chemotherapy with autologous stem cell rescue, radiation, and GD2-based immunotherapy for the high-risk group (Figure 1) [10,13,15,16].

Much attention has been directed toward immune checkpoint inhibitors (ICIs) as the prototypical anticancer immunotherapy approach since the FDA approval of the anti-CTLA4 monoclonal antibody (mAb) ipilimumab in 2011 for the treatment of melanoma. ICIs targeting CTLA4 and PD1/PD-L1 have demonstrated efficacy across a range of adult solid tumors [17,18]. However, the efficacy for childhood cancers, including NBL, has remained disappointing [19]. This is thought to be due to a series of factors that are unique to childhood tumors, including the lack of neoantigens, a highly immunosuppressive TME, and tumor-supportive neovascularization [20]. As such, there remains a pressing need to identify the barriers to successful application of existing immunotherapeutic approaches for NBL and pediatric solid tumors in general, as well as identifying opportunities for novel immune-modifying therapies that may be uniquely suited to deal with the challenges posed by these tumor types. 

In this review, we focus on the immune cellular components of the TME in NBL, current GD2-based immunotherapy and combinations, as well as recent advancements and emerging novel strategies. To identify publications for inclusion in our review, we initially searched PubMed for the combination of keywords “neuroblastoma” and “immunotherapy”. Individual publications were selected based on direct relevance to the focus of this review. Reference lists for each included paper were also reviewed to identify additional publications that were relevant to our focus and would inform our conclusions. We anticipate that this review will offer readers a platform for critically evaluating the current potential for immunotherapy in NBL.

## 2. Barriers to Effective Immunotherapy in NBL

**Figure 2:**
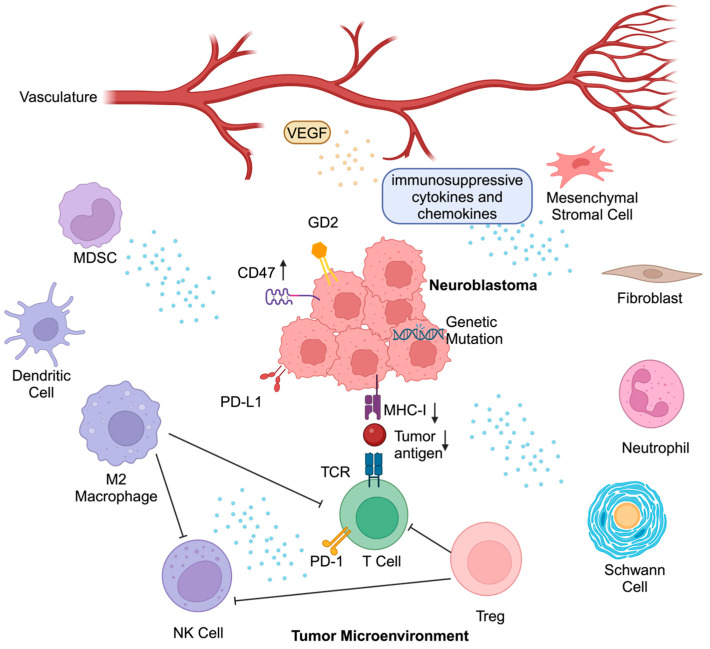
Barriers imposed by neuroblastoma (NBL) for effective immune-based treatments. Multiple mechanisms contribute to an immunosuppressive tumor microenvironment and function as barriers to effective adoption of immune-based treatments in NBL. These include overexpression of the “don’t eat me” signal CD47, low mutational burden and thus low neoantigen load, which prevents the effective recruitment of tumor-infiltrating lymphocytes (TILs) and limits effective antigen presentation by macrophages and dendritic cells (DCs). Moreover, the NBL tumor microenvironment (TME) is also infiltrated by a variety of anti-inflammatory immune cells, including tumor-associated macrophages (TAMs), myeloid-derived suppressor cells (MDSCs), and secreted immunosuppressive factors, including TGFβ, indoleamine 2,3-dioxygenase (IDO), Arg-2, HMGB-1, and other pro-angiogenic factors. Regulatory T cells (Tregs) in the NBL TME further interfere with the effector T cells and natural killer (NK)-cell function. Moreover, non-immune cells such as mesenchymal stromal cells (MSCs), fibroblasts, Schwann cells, and endothelial cells each play unique roles in contributing to an immunosuppressed TME in NBL. This figure was created with BioRender.com.

NBL, like other childhood solid tumors, has a low immunogenicity profile, which is exemplified by low expression of major histocompatibility complex I (MHC-I), overexpression of the “don’t eat me” signal CD47, and a low tumor mutational burden, leading to a low neoantigen load [21,22,23]. These features work against the effective recruitment of tumor-infiltrating lymphocytes (TILs), resulting in an immunosuppressed TME [24]. Moreover, the NBL TME is predominantly composed of anti-inflammatory immune cells, such as tumor-associated macrophages (TAMs) and myeloid-derived suppressor cells (MDSCs), both of which secrete multiple immunosuppressive factors [6,25,26,27]. In this section, we will individually address the following factors that have thus far served as barriers to effective immunotherapeutic approaches in NBL: (1) immune cells and cytokines, (2) non-immune cells, (3) tumor neo-vasculature, and (4) the low intrinsic immunogenicity of NBL tumor cells (Figure 2).

### 2.1. Immune Cells and Cytokines

#### 2.1.1. Macrophages

Heterogeneous myeloid populations consisting of immature cell types with immunosuppressive characteristics are typically found in the TME of most solid tumors. These myeloid populations can be further separated into two major populations, TAMs and MDSCs, based their expression of selected cell surface markers [28,29]. TAMs have recently become a focus of research efforts in many different tumor types, as a growing body of evidence suggests that they play a central role in tumor progression and coordinating the immune cellular populations present within the TME [30]. The density of TAMs within the TME correlates with prognosis and progression in NBL [25,31]. Interestingly, TAMs can upregulate oncogenic MYC expression in non-MYCN amplified NBL cells, contributing to tumor progression [32].

Additional evidence indicates that TAMs can promote NBL growth through stimulating the secretion of angiogenesis-promoting factors such as vascular endothelial growth factor (VEGF), platelet-derived growth factor (PDGF), transforming growth factor-β (TGF-β), matrix metalloproteinase-2 (MMP2), MMP9, and hypoxia-inducible factor (HIF-2α) [33,34]. TAMs further support tumor cell invasion via increased interactions with CXCR2 on NBL cells [35]. TAMs also regulate the crosstalk between other immune cell populations, indirectly affecting tumor progression, for example between regulatory T cells (Tregs) and MDSCs. TAMs directly inhibit effector immune cell types including natural killer (NK) cells and cytotoxic T cells [36]. TAM-mediated inhibition of effector NK cells is one mechanism that may limit efficacy of anti-GD2 therapies in NBL, despite the demonstrated success of this approach [37,38]. TAMs are also able to increase cancer-associated fibroblast (CAF) proliferation and invasion [39], and there is evidence that the interactions between CAFs and TAMs contribute to NBL development [35]. These data collectively demonstrate the critical role of TAMs in NBL.

#### 2.1.2. MDSCs

MDSCs are a heterogeneous immune cell population that are generally considered to contribute to immunosuppression of the TME through direct and indirect modulation of multiple other immune cell types such as T cells, NK cells, antigen-presenting cells (APCs), and B cells [29,40] and through secretion of tumor-promoting factors such as reactive oxygen species (ROS), Arg-1, and TGF-β. In a murine model of NBL, injection of MDSCs accelerated NBL growth [41]. MDSCs are also increased in the circulating blood and local tumors in NBL patients [41].

In mice, MDSCs can be further divided into two different subpopulations using the cell surface markers Ly6G and Ly6C: the Ly6C+ Ly6G−/low mononuclear MDSCs (M-MDSCs) and the Ly6G+ Ly6C-/low granulocytic or polymorphonuclear MDSCs (PMN-MDSCs). M-MDSCs are generally considered to be more suppressive than PMN-MDSCs in mice [42]; however, the functional differences between these two subpopulations is less well defined in humans [43,44].

#### 2.1.3. NK Cells

NK cells are a first line of defense against tumor development, given their ability to recognize and attack cancer cells without the need for antigenic stimulation. Consistent with their antitumor function, clinical data demonstrate that lower numbers of NK cells and lower levels of the NK-activating cytokine IL-15 predict poorer outcomes in *MYCN* non-amplified NBL [45]. Moreover, activated NK cells can inhibit the local growth of NBL, slow down the process of metastasis, and attenuate the associated immunosuppression within the TME. miR-168 exosomes derived by activated NK cells can also inhibit the growth and immunosuppressive effects of NBL [46]. Therefore, it is of great interest to investigate and understand the role of NK cells in NBL to improve the therapeutic potency [47].

#### 2.1.4. T Cells

T cells have long been observed in human NBL for the correlation between their density in the TME and the severity of disease course [48,49]. Despite this observation, there are very few tumor-infiltrating T cells in NBL [50]. T cells that do manage to infiltrate the TME are further suppressed by soluble factors present in the TME, such as transforming growth factor-β (TGF-β), interleukin (IL)-10, and galectin-1 [51,52,53,54]. Interestingly, there are more CD8+ T cells than CD4+ T cells within the infiltrated T cells [55]. Solid tumor infiltrating T cells in general will transform to a regulatory T cell (Treg) phenotype over time. Tregs suppress immune activation and contribute to tumor immune escape through multiple mechanisms. Examples include the following: (1) Tregs secrete a number of inhibitory cytokines, including TGF-β, IL-35, and IL-15; (2) Tregs can produce granzyme and perforin, which induce apoptosis in effector cells; (3) Tregs prevent co-stimulation through CD28 on effector T cells to APCs through CTLA-4 [56,57,58]; and (4) Tregs act as a molecular sink for secreted factors in the TME through expression of receptors for inflammatory cytokines, which in turn serves to impede the activation of effector cells [59,60,61]. Early studies in mouse NBL show that the depletion of Tregs potentiates other immunotherapies [62,63]. Moreover, activated T cells with constitutively active protein kinase B, also known as Akt kinase, illustrated resistance to multiple tumor-associated suppressive mechanisms in NBL [64]. These data support that T cells as a population represent a possible therapeutic target for immunotherapeutic approaches in NBL.

### 2.2. Stromal Cells

#### 2.2.1. Cancer-Associated Fibroblasts (CAFs)

CAFs are among the most abundant cell types in tumor stroma and contribute to tumorigenesis, metastasis, and immunosuppression across different solid tumors. CAFs form a dense extracellular matrix creating physical barriers for effective treatments [65]. CAFs are abundant in NBL [66], which may be due to a high level of TGF-β in NBL [67] or Schwann cell infiltration, a unique histologic characteristic of NBL [68]. TGF-β activates CAFs in other tumor types and is also highly prevalent in the NBL TME. A recent study demonstrated that CAFs correlate with worse outcomes in stage 4 *MYCN* amplified NBL [68]. As discussed in the previous section, CAFs and TAMs are found in close physical proximity and interact with each other. CAFs can recruit TAMs and transform TAMs to be more M2-like and therefore more immunosuppressive [35,69,70].

#### 2.2.2. Mesenchymal Stromal Cells (MSCs)

Similar to CAFs, MSCs are abundant in many cancer types and are capable of promoting tumor proliferation, invasion, and metastasis in NBL [71,72]. High levels of C-X-C motif chemokine ligand 12 (CXCL12) secreted by MSCs are pivotal in promoting NBL invasion and metastasis [71,73]. MSCs in bone marrow metastasis can further promote metastasis through secretion of CXCL13 [74]. Moreover, MSC and NBL tumor cell crosstalk promotes a more tumorigenic and immunosuppressive microenvironment [75]. MSCs can interfere with antibody-dependent cell cytotoxicity (ADCC) through TGFβ signaling, which creates challenges for immunotherapy and in particular GD2-based approaches [76]. Antioxidant enzymes secreted by MSCs also contribute to the tumorigenicity of the TME by reducing the prevalence of damaging reactive oxygen species [39].

#### 2.2.3. Schwann Cells

Schwann cells constitute a unique stromal cell population in NBL whose presence is inversely correlated with histologic severity of disease. The mechanisms by which Schwann cells coordinate the immune state of the NBL TME are complex. For example, the progenitors of Schwann cells are able to transform into chromaffin-like NBL tumor cells [77]. In addition to directly leading to tumorigenesis, Schwann cells can influence NBL tumor cell differentiation. Increased neuronal differentiation was found when NBL cells were cultured in Schwann cell-conditioned medium [78]. Further studies revealed that nerve growth factor (NGF) and epidermal growth factor-like protein 8 (EGFL8) are able to induce neuroblastoma differentiation and hence inhibit tumor proliferation [79,80]. This anti-tumor effect may be utilized if replaced with the recombinant counterparts. Furthermore, induced autophagy of the Schwann cells in the NBL TME will promote tumor proliferation [81]. These findings highlight how Schwann cells are a unique contributor to NBL and could represent a target for therapy as more becomes understood regarding their role as a regulator of the TME in NBL.

### 2.3. Vasculature

VEGF drives angiogenesis in mouse and human NBL [82,83] and the resulting vascular complexity correlates with the aggressiveness in human NBL [84]. Moreover, the convoluted nature of tumor neo-vasculature in solid tumors creates another physical barrier for efficacious drug delivery and thereby hinders therapeutic efficacy in general [85,86]. Targeting the tumor vasculature of NBL may serve to enable the efficacy of other therapeutic agents in addition to the potential for direct effects on tumor cells.

### 2.4. NBL Tumor Cells and Their Intrinsic Low Immunogenicity

NBL tumor cells have an intrinsic low immunogenicity. This is in large part due to the low expression of major histocompatibility complex-I (MHC-I) molecules on NBL cells, which impedes efficient antigen presentation to T cells and the related cytotoxic cellular machinery [22,23,87]. On the other hand, Fas ligand (FasL) is expressed on NBL cells and neutralizes infiltrated T cells, which not only makes the TME more permissive to native tumor growth, but also adds complexity to the design and development of potential immunotherapies [88]. Moreover, the *MYCN* oncogene itself can directly limit T cell infiltration as well as attenuate NK cell activation, which combine to support immune evasion of high-risk NBL [24,89,90,91].

NBL cells can also impair the antitumor immune functions of multiple effector cells through secretion of arginase (Arg)-2 [6,92], high mobility group box-1 (HMGB1) [93], and other pro-angiogenic factors [94]. For example, HMGB1 secreted by NBL tumor cells can induce Schwann cell autophagy and support NBL proliferation [81]. NBL tumor cells also secrete cytokines. In mouse NBL, macrophage inhibitory factor (MIF) was shown to be produced by the tumor cells which lead to the silencing of effector T cells [95]. In contrast, low production of monocyte chemoattractant protein-1 (MCP-1) in NBL also attenuates the tumor tropism and thus interferes with therapeutic success [96].

## 3. Current Immunotherapies for NBL

### 3.1. Therapeutic Strategies to Target Cancer Cells

#### Anti-GD2 Antibodies and the Derived Therapies

Currently, the only immunotherapy to successfully be adopted into widespread clinical practice is monoclonal antibody targeting the disialoganglioside GD2. GD2 is highly expressed in NBLs across disease stages with limited expression in selected normal cells such as peripheral sensory neurons [97,98,99,100]. GD2 is a promising tumor-associated antigen (TAA) for targeted immunotherapy given its limited expression pattern to tumor cells of all stages with limited normal tissue expression. Multiple rounds of development and molecular re-design led to the most widely used form of GD2-targeting antibody, dinutuximab (CH14.18/SP2/0), a chimeric IgG1 anti-GD2 mAb [101]. When combined with granulocyte-macrophage colony-stimulating factor (GM-CSF) and IL-2, dinutuximab demonstrated excellent efficacy and significantly prolonged the overall survival in high-risk disease [102].

Although dinutuximab is the only anti-GD2 mAb effective for standard therapy, various trials are underway investigating the application of immunotherapy involving anti-GD2 monoclonal antibodies in different contexts and approaches (NCT02258815, NCT01701479, NCT01767194, NCT02308527, NCT03794349, NCT01717554, NCT02914405). Multiple studies have shown that addition of dinutuximab beta to the chemotherapy combination significantly increases event-free survival and overall survival compared to chemotherapy treatment alone [103,104]. Interestingly, a phase III clinical trial found that subcutaneous administration of IL-2 alongside dinutuximab beta treatment did not yield improved outcomes [103,105]. Nonetheless, the combination of GM-CSF with anti-GD2 antibodies in both the consolidation/maintenance phase and the relapsed/refractory disease setting in NBL patients were shown to be safe and efficacious [102,106].

Despite the favorable expression pattern of GD2 on NBL tumor cells, neuropathic pain remains a major problem and is the primary dose-limiting toxicity associated with this therapeutic approach [107]. This is due to the ability of anti-GD2 mAb to bind to GD2+ myelin sheaths of nerve fibers, which necessitates mAb dose-reduction and the use of narcotic pain medications. Hence, effort has been applied to identify other cell-surface antigens as well as engineering existing anti-GD2 therapies to overcome neuropathic pain. Some recent efforts include targeting the B7-H3 checkpoint molecule. B7-H3 is overexpressed on NBL and other pediatric solid tumor cells, with restricted expression on normal tissues, and thus has the potential to limit the incidence and severity of neuropathic pain [108,109]. More recent reports suggest that this problem has largely been overcome as recent phase I/II trials report lower rates of grade 3 or 4 pain, and almost no patients stop therapy for this reason [110].

### 3.2. Therapeutic Strategies to Target Immune Cells

#### 3.2.1. Recruitment

As described earlier, MDSCs and TAMs are recruited to the NBL TME and contribute to tumor progression. One potential therapeutic approach would be interference with chemoattractant molecular systems to inhibit the accumulation of these suppressive myeloid cells. Indeed, targeting CSF-1/CSF-1R axis slows NBL progression and increases the efficacy of ICIs and chemotherapy in an NBL mouse model [111,112,113]. Alternatively, attenuating tumor inflammation in theory would also reduce the prevalence of suppressive innate cells. In a mouse NBL model, it was shown that low-dose aspirin treatment effectively delays tumor progression with a reduced size of tumor burden, with fewer tumor-infiltrating MDSCs and TAMs [114].

#### 3.2.2. Depletion

Depletion of the myeloid cells that have already managed to infiltrate the NBL TME is an alternative strategy to mitigate disease progression. Application of anti-CD11b antibody therapy in NBL-bearing mice increased the antitumor efficacy of anti-GD2 antibody therapy [115]. Similarly, targeting myeloid cells augments the antitumor efficacy of anti-PDL1 therapy in high-risk NBL [107]. More recently, MDSCs have been intensively studied in various immunotherapeutic approaches for NBL. Elimination of MDSCs is an effective method to improve the antitumor effect of immunotherapy for NBL in a mouse model and to increase the T cell infiltration in TME [116,117]. Similarly, a reduction in MDSCs strengthened the immune response induced by anti-GD2 antibody [115].

#### 3.2.3. Repolarization

Suppressive myeloid cells can also be repolarized to be immunostimulatory via various therapeutic agents. One strategy is to inhibit histone deacetylase (HDAC) proteins with vorinostat. Indeed, treatment with vorinostat repolarized TAMs and MDSCs in an NBL model [118]. Furthermore, vorinostat also synergizes with anti-GD2 mAbs [118], exhibiting the potential to be included with other combination therapies.

Another application of the concept of repolarization of innate immune cells is polyphenon E, a therapeutic agent able to reshape MDSCs to a more granulocytic phenotype, which is shown to attenuate the immunosuppression in NBL [41]. Targeted IL-2 therapy has been shown to effectively activate NK cells and eradicate NBL metastasis to the bone marrow [47]. Moreover, IL-2 in combination with other compounds such IL-18 [119], fractalkine [120] or lenalidomide [38] can reactivate NK cells and thus to improve efficacy against NBL. Other agents including, but not limited to, IFN-γ [121], PGE2 inhibitor [69], and CXCR4 antagonist [122] have all been shown to repolarize TAM populations in the NBL TME, presenting great potential as effective treatments for NBL.

#### 3.2.4. Immune Checkpoint Blockade

The emergence of ICI therapy has led to a paradigm shift for cancer immunotherapy in the past decade. ICI targeting programmed cell death protein-1 (PD-1)/programmed cell death-ligand 1 (PD-L1), and cytotoxic T-lymphocyte associated protein-4 (CTLA-4)/B7 are the most widely studied and utilized immunotherapies to reverse immunosuppression by blocking specific cell surface signals that suppress T cell responses.

Even though ICIs targeting PD-1 (Nivolumab, Pembrolizumab, and Cemiplimab), PD-L1 (Atezolimumab, Durvalumab, and Avelumab), and CTLA-4 (Ipilimumab) have been effective against metastatic melanoma, renal cell carcinoma, head and neck cancers, and non-small lung cancer [123], they have failed to demonstrate efficacy in NBL [19,124,125]. On the other hand, combinations of ICIs yielded promising results. The combination of anti-PD-1 and anti-CTLA-4 with cyclophosphamide significantly prolonged survival in comparison with those treated with ICI monotherapy in a murine model [37]. Moreover, the combination of anti-PD-1 and anti-CTLA-4 with whole tumor cell vaccination exhibited impressive results and was able to counter the adaptive immune resistance to elicit strong tumor efficacy in a mouse NBL model [126]. These results show the promise of combinatorial strategies for checkpoint inhibition in NBL.

### 3.3. Therapeutic Strategies to Target Other Cells

#### 3.3.1. Cytokines and Stromal Cells

Application of CXCR4 antagonist successfully improved the efficacy of dendritic cell vaccine in a mouse NBL model [122]. Anti-CD105 Ab targets a component of the TGFβ receptor complex leading to functional inhibition of TGFβ receptor signaling and improves anti-GD2-induced ADCC [76]. On the other hand, studies have shown that it is feasible to utilize the homing ability of MSCs to form a new drug-delivery platform carrying either oncolytic viruses [127] or immunostimulant cytokines [121].

#### 3.3.2. Vasculature

Inhibiting and reversing angiogenesis has shown some promise in NBL. Multiple levels of angiogenesis inhibition, including Notch suppression, prostaglandin E synthase inhibition, endothelial growth blocking, and targeting hypoxia-inducible factors (HIFs), are all able to suppress NBL tumor growth [69,128,129]. Application of anti-VEGF antibody induced vascular remodeling and led to more efficient delivery of chemotherapy, resulting in higher anti-tumor drug efficacy in NBL [85].

## 4. Recent Advances and Future Directions of Immunotherapies for NBL

Drawing from clinical and biological prognostic indicators like the standard International NBL Risk Group staging, age, histological classification, tumor differentiation grade, MYCN status, presence of 11q abnormalities, and tumor cell ploidy, the Children’s Oncology Group (COG) has devised a risk stratification framework for NBL (NBL). This classification system segments NBL cases into distinct risk groups: very low, low, intermediate, and high risk [4]. For most low-risk NBL patients, surgery suffices as curative treatment, while chemotherapy is reserved for specific cases [12]. Intermediate-risk NBL patients receive reduced chemotherapy compared to earlier trials, with survival rates nearing 100%, contrasting sharply with the below 50% rates seen in high-risk cases [16,130,131,132].

Given that most patients with high-risk NBL either do not respond to initial treatments or experience relapses within two years, which typically involve multiple stages of chemotherapy, surgery, stem cell transplantation, radiotherapy, and subsequent therapies, the importance of focusing on precision medicine becomes critical for adapting to individual cases [16,133,134,135]. In this section of the review, we will focus on several aspects of targeted therapy, molecular biomarkers, and immunotherapy for NBL.

### 4.1. Targeted Drugs

#### 4.1.1. Anaplastic Lymphoma Kinase (ALK) Inhibitors

ALK, a member of the insulin receptor superfamily, plays a significant role in numerous cancers as an oncogene, characterized by mutations including copy number variations, amplifications, and point mutations [136,137]. Most NBLs express the full-length ALK receptor [136,138] and high expression of ALK correlated with poor prognosis [139,140]. In NBL, activating mutations are responsible for 6–10% of cases, with an additional 3–4% carrying high-risk ALK mutations. The primary variation sites are F1174, F1245, and R1275, with the latter compromising 85% of all ALK-positive tumors [2,141]. The prevalence of ALK gene mutations escalates in recurrent NBL, occurring in roughly 20% of cases, and abnormal copy numbers in high-risk NBL cohorts correlates significantly with disease-related mortality [142,143,144]. Targeting the ALK receptor has been identified as one of the most promising and specific approaches for NBL therapy [145,146,147].

Crizotinib, a first-generation ALK inhibitor, has been extensively investigated for its efficacy in patients with relapsed or refractory cancer, particularly demonstrating significant success in treating children with anaplastic large cell lymphoma and inflammatory myofibroblastic tumors [148]. Early clinical trials showed limited clinical efficacy, with responses observed only in patients carrying the somatic ALKR1275Q mutation but not in those with ALK amplification or the ALKF1174L mutation, indicative of resistance to certain ALK hotspot mutations [145,149,150]. This resistance stems from heightened ATP-binding affinity resulting from ALK residue mutations despite the presence of potentially sensitive mutations, necessitating higher doses of ALK inhibitors for mitigation. Nonetheless, a recent clinical trial encountered analogous challenges, illustrating limited efficacy primarily due to its failure to achieve sufficiently high concentrations to counteract this ATP affinity competition [145]. Combination therapies are being tested to augment crizotinib’s efficacy by combining it with chemotherapy agents typically used in high-risk NBL, leading to a synergistic effect. This synergy is a key reason why crizotinib has been included in the treatment plan for high-risk NBL patients with ALK mutations in a phase 3 clinical trial (NCT03126916) [151].

Second- and third-generation ALK inhibitors such as eritinib, lorlatinib, brigatinib, alectinib, and repotrectinib have shown enhanced therapeutic efficacy in NBL cases with ALK mutations, albeit inducing complete responses in only a few patients [149,152,153,154,155,156]. Encouragingly, lorlatinib has been thoroughly studied, showing promise as an alternative to crizotinib. It can be utilized independently or alongside chemotherapy to address ALK-driven refractory or recurrent high-risk NBL safely and effectively [157,158,159]. Additionally, lorlatinib also exhibits potential in managing adult-onset NBL, with 69% of patients demonstrating durable primary response and almost all patients remaining progression-free at a median of 19 months [160,161]. To overcome resistant mutations or mutations that develop to impede the effectiveness of various drug inhibitors, some have focused on creating antibody–drug conjugates (ADCs) consisting of a gene-recognizing antibody and a toxin that kills the gene-expressing cells, demonstrating some efficacy in pre-clinical models [162].

#### 4.1.2. Small Extracellular Vesicles

Tumor-derived small extracellular vesicles (sEVs) have become pivotal players in influencing the effectiveness of immunotherapy [163]. Ranging from 30 to 150 nm, these vesicles are released by nearly all cell types either through outward budding of the plasma membrane or direct fusion of multivesicular bodies with the plasma membrane [164]. Remarkably, sEVs carry biologically active molecules with the capacity to alter the extracellular milieu and immune responses by interacting with immune effector cells and suppressing the host immune system [165,166]. As described earlier, NK cells serve as primary effectors in anti-GD2 immunotherapy, employing ADCC to eliminate NBL cells; however, sEVs impair ADCC by inhibiting binding of antibodies to tumor cells and inducing NK cell exhaustion [167,168,169,170]. In vitro pharmacological inhibition of sEV secretion with tipifarnib sensitizes NBL tumors to immunotherapy [171]. Tipifarnib is an FDA approved farnesyltransferase inhibitor that has shown high clinical success (objective response rate of 55%) in patients with recurrent and/or metastatic head and neck squamous cell carcinoma [172].

#### 4.1.3. Aurora Kinase A (AURKA) Inhibitors

AURKA represents a therapeutic focal point across several malignant tumors, with heightened expression levels correlating with the state of *MYCN* amplification and diminished overall survival in NBL patients [173,174]. Earlier studies showed the potential of AURKA inhibitors to interfere with *MYCN* stability and tumor regression [175,176]. Clinical trials on alisertib and erbumine demonstrated promising pharmacokinetic-pharmacodynamic associations in preclinical and adult studies [177]. Alisertib exhibited good activity in pediatric xenograft models, but its objective remission rates in pediatric patients with refractory or recurrent solid tumors or acute leukemia were less than 5% [178,179,180]. Clinical trials evaluating the combination of AURKA inhibitor, alisertib, and chemotherapy agents, irinotecan and temozolomide, exceeded anti-tumor activity of each treatment alone [181,182]. Interestingly, research suggests that elevated aurora kinase B (AURKB) levels in NBL cells are strongly associated with acquired resistance to carboplatin, a prevalent chemotherapy agent for NBL treatment, due to upregulation of the AURKB– extracellular signal-regulated kinase (ERK) axis [183]. Thus, inhibition of aurora kinase (AURK) presents a promising therapeutic approach in combating carboplatin resistance in NBL patients.

### 4.2. Immunotherapy and Novel Therapeutics

Immunotherapy shows significant potential in enhancing survival rates across various adult cancers through immune checkpoint inhibition, antibody-mediated therapy, and adoptive T cell therapy. Recently, most noteworthy are the improved survival outcomes in high-risk NBL patients with the introduction of anti-GD2 therapy, underscoring the promise of immunotherapy in pediatric oncology [184,185]. Providing immunological memory against the tumor to prevent disease relapse would be beneficial but cannot be accomplished without overcoming the low immunogenicity and suppressive TME of NBL discussed above [23,186].

#### CAR-T Cell Therapies

Recently, T cells modified to express chimeric antigen receptors (CARs) have become a prominent addition to the expanding array of immunotherapies for cancer treatment. CARs combine the epitope specificity of a monoclonal antibody with the cytolytic potential of an activated T cell, enabling the targeting of tumors expressing the corresponding cell surface antigen independently of major histocompatibility complex (MHC) presentation. CAR T cells have shown notable efficacy in combating B cell malignancies; however, their clinical impact in solid tumors remains less pronounced [187,188,189]. The latest clinical trials have narrowed in finding the appropriate amount of autologous third generation GD2-CAR T cells expressing the inducible caspase 9 suicide gene that can be safely administered to patients and recorded significant decrease in tumor size as well as a 3-year overall survival of 60% [190,191]. Nonetheless, T-cell persistence, target antigen selection, and the immunosuppressive TME remain challenges and areas for improvement.

Similarly, antibodies targeting the extracellular domain of ALK have shown to induce cell death in NBL cell lines, suggesting the potential for ALK to serve as a feasible CAR T cell target [192]. Pre-clinical studies have shown inconsistent results; some demonstrated efficacy against ALK wild-type or mutated NBL models while others did not, attributing lack of success to low surface receptor density [162,193]. Recently, Bergaggio et al. presented the development of ALK.CAR-T cells targeting NBL when ALK was expressed at high levels, while in NBL cases characterized by low ALK density, ALK inhibitors (lorlatinib) notably enhanced the tumor-targeting capabilities of ALK.CAR-T cells [194].

Plasticity of ALK expression in tumors may be overcome through improved combinations such as cyclic administration of ALK tyrosine kinase inhibitors (TKIs), higher doses, or strategies to improve ALK stabilization on the cell surface [194,195]. A similar concept could be applied in restoring G2D expression on NBL cells transitioning to mesenchymal by inhibiting EZH2 and preventing GD2 downregulation, therefore improving monoclonal anti-GD2 therapy. Lastly, combining the activity of ALK.CAR-Ts and GD2.CAR-Ts in a dual CAR construct could combine the broad activity of GD2.CAR-Ts with the specificity of ALK.CAR-Ts, resulting in a strong synergistic effect.

Vα24-invariant natural killer T (NKT) cells are known for their robust anti-tumor effects in murine tumor models and are associated with positive patient outcomes in cancer [53,196,197,198]; therefore, new studies are leveraging the innate and adaptive immune-modulating properties of NKT cells as an alternative platform for CAR-redirected immunotherapy [199,200]. To increase the frequency of NKT cells in peripheral blood mononuclear cells (PBMCs) and reach clinical-scale NKT cell expansion, NKT cells were engineered to co-express a GD2 CAR with IL-15 in children with relapsed or resistant NBL (NCT03294954) [201]. These constructs were found to have no dose-limiting toxicities and were able to mediate objective responses in patients with NBL that could be further enhanced by targeting BTG1 [202].

## 5. Conclusions

**Figure 3:**
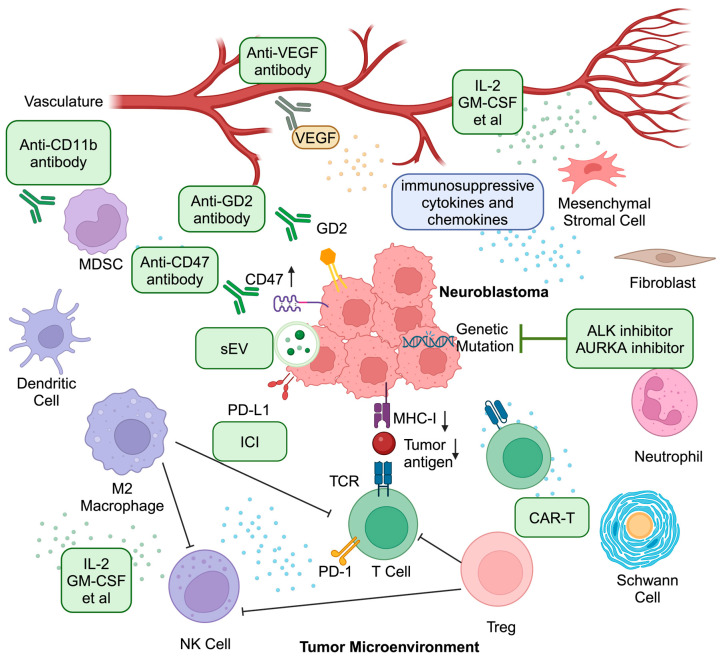
Current and emerging therapies to potentially overcome the barriers imposed by neuroblastoma (NBL) for effective treatments. This simplified scheme includes current and emerging immunotherapies targeting the barriers imposed by NBL and thus leading to promising potential for effective treatments. ALK inhibitors and AURKA inhibitors would target the NBL tumor cells directly. Likewise, sEVs have the potential to increase detection of NBL by immune cells and elicit effective killing mechanisms. Cytokine-based therapies could overcome the immunosuppressive cytokines and chemokines to shift local inflammation from tumor-supportive to tumor-killing. Depletion strategies utilizing anti-CD11b may mitigate the immunosuppression in the tumor microenvironment. Anti-GD2 antibodies can enhance the antibody-dependent cell mediated cytotoxicity and tumor phagocytosis. Similarly, anti-CD47 antibodies could enhance phagocytosis of tumor cells. Though ICIs do not show significant benefits as single-agent treatments, the combination of ICIs with other immunotherapies may enhance their efficacy in NBL. Artificial expression of CARs on autologous T cells or invariant NK T cells may further boost adaptive immunity against NBL. This figure was created with BioRender.com.

Even though almost all efforts to develop immunotherapies for NBL have focused on targeting GD2, several other differentially expressed antigens have more recently gained attention. Significant progress has been made on other targets such as GPC2 and ALK. Novel therapies including CAR-T, engineered antibodies, nanomedicines, and cancer vaccines have also attracted research attention and are undergoing clinical trials (summarized in Figure 3). Promising immunotherapeutic options in development for NBL are highlighted in Table 1. In addition, encouraging progress is being made in nanomedicine, epigenetic regulators, cancer vaccines, and prognostic and diagnostic marker development. Taken together, a combination of systematic preclinical investigations and early-phase clinical trials will be needed to advance the utilization of combination immunotherapy for NBL. In vitro and in vivo preclinical models would be anticipated to define novel immune mechanisms of NBL progression and treatment resistance that may offer therapeutic vulnerabilities not previously recognized. It is our opinion that these preclinical investigations should be the near-term focus for the field in general, to maximize the probability of identifying novel mechanisms and therapeutic options to test in the clinic in the next generation of early-phase clinical trials.

## Figures and Tables

**Table 1:** Types of representative immunotherapies for NBL under investigation or in clinical trials.

Type	Rationale	Example	Relevant Trial Identifier/Reference
Anti-GD2 mAb	Augmenting the immunotherapeutic effects of anti-GD2 mAb in combination with chemotherapies, and/or other immunotherapies	Dinutuximab + IL-2 + GM-CSF	[102,107]
		Dinutuximab + lenalidomide + retinoic acid	NCT01711554
		Dinutuximab beta + conventional chemotherapy	NCT01701479
		Dinutuximab + magrolimab (anti-CD47)	NCT04751383
		Dinutuximab + lenalidomide + expanded autologous NK cells	NCT02573896
		Hu14.18-IL2 + expanded haploidentical NK cells	NCT03209869
		Dinutuximab beta + IL-2	NCT02258815
		Dinutuximab beta + anti-CD11b	[115]
		14.G2a + vorinostat	[118]
		Dinutuximab beta + nivolumab (anti-PD1) + ^131^I-MIBG	NCT02914405
Anti-B7-H3	Targeting NBL-tumor cells with restricted expression on normal cells, reducing major side effects	Anti-B7-H3 mAb	[108]
		Bispecific antibody of GD2 and B7-H3	[110]
ALK inhibition	ALK mutations correlated with poor NBL prognosis	Crizotinib	NCT03126916
		Lorlatinib with chemotherapy	[158]
AURKA inhibition	Heightened expression of AURKA correlated with *MYCN* amplification and overall survival in NBL patients	SK2188	[176]
		Alisertib + Irinotecan + Temozolomi	[181,182]
ICI	Countering adaptive immune resistance	Anti-PD-1 and anti-CTLA-4 with cyclophosphamide	[37]
		BLZ945 (CSF-1R inhibitor) + PD-1/PD-L1 blocking antibodies	[112]
		Anti-PD-1 and anti-CTLA-4 with whole tumor cell vaccination	[126]
CAR	CARs combine the epitope specificity of a monoclonal antibody with the cytolytic potential of an activated T cell, enabling the targeting of tumors expressing the corresponding cell surface antigen independently of MHC presentation	Anti-GD2 CAR-NKT cell	NCT03294954
		CAR T cells targeting B7-H3	[109]
		NKG2D.ζ-NK cells + CAR-T cells	[117]
		ALK.CAR-T cell + lorlatinib	[194]
Angiogenesis inhibition	Vascular complexity correlates with the aggressiveness in human NBL	Bevacizumab (anti-VEGF) + chemotherapy	[85]
		mPGES-1 inhibitor	[69]

## Data Availability

All relevant data are included within the paper.

## Abbreviations

ADC	antibody–drug conjugate
ADCC	antibody-dependent cell cytotoxicity
ALK	anaplastic lymphoma kinase
Arg-2	Arginase-2
AURK	aurora kinase
AURKA	aurora kinase A
AURKB	aurora kinase B
CAF	cancer-associated fibroblast
CAR	chimeric antigen receptor
COG	Children’s Oncology Group
DC	dendritic cell
ERK	extracellular signal-regulated kinase
EGFL8	epidermal growth factor-like protein 8
FasL	Fas ligand
GM-CSF	granulocyte-macrophage colony-stimulating factor
HIF	hypoxia-inducible factor
HMGB1	high mobility group box-1
HR NBL	high-risk NBL
ICI	immune checkpoint inhibitor
IL	interleukin
mAb	monoclonal antibody
MCP	monocyte chemoattractant protein
MDSC	myeloid-derived suppressor cell
MHC	major histocompatibility complex
MIF	macrophage inhibitory factor
M-MDSC	mononuclear MDSC
MSC	mesenchymal stromal cell
NBL	neuroblastoma
NGF	nerve growth factor
NK cell	natural killer cell
NKT	natural killer T cell
PBMC	peripheral blood mononuclear cell
PDGF	platelet-derived growth factor
PMN-MDSC	polymorphonuclear MDSC
ROS	reactive oxygen species
sEV	small extracellular vesicle
TAA	tumor-associated antigen
TAM	tumor-associated macrophage
TGF	transforming growth factor
TIL	tumor-infiltrating lymphocyte
TME	tumor microenvironment
TKI	tyrosine kinase inhibitor
Treg	regulatory T cell
